# Novel nanoadjuvants balance immune activation with modest inflammation: implications for older adult vaccines

**DOI:** 10.1186/s12979-023-00349-5

**Published:** 2023-06-21

**Authors:** Kathleen A. Ross, April M. Tingle, Sujata Senapati, Kaitlyn G. Holden, Michael J. Wannemuehler, Surya K. Mallapragada, Balaji Narasimhan, Marian L. Kohut

**Affiliations:** 1 Nanovaccine Institute, Iowa State University, Ames, IA 50011 USA; 2 Immunobiology, Iowa State University, Ames, IA 50011 USA; 3 Chemical and Biological Engineering, Iowa State University, Ames, IA 50011 USA; 4 Veterinary Microbiology and Preventive Medicine, Iowa State University, Ames, IA 50011 USA; 5 Kinesiology, Iowa State University, Ames, IA 50011 USA

**Keywords:** Aged, Nanovaccines, Polyanhydrides, Pentablock copolymer micelles, Cyclic dinucleotides

## Abstract

**Background:**

Age-associated impairments of immune response and inflammaging likely contribute to poor vaccine efficacy. An appropriate balance between activation of immune memory and inflammatory response may be more effective in vaccines for older adults; attempts to overcome reduced efficacy have included the addition of adjuvants or increased antigenic dose. Next generation vaccine formulations may also use biomaterials to both deliver and adjuvant vaccine antigens. In the context of aging, it is important to determine the degree to which new biomaterials may enhance antigen-presenting cell (APC) functions without inducing potent inflammatory responses of APCs or other immune cell types (e.g., T cells). However, the effect of newer biomaterials on these cell types from young and older adults remains unknown.

**Results:**

In this pilot study, cells from young and older adults were used to evaluate the effect of novel biomaterials such as polyanhydride nanoparticles (NP) and pentablock copolymer micelles (Mi) and cyclic dinucleotides (CDN; a STING agonist) on cytokine and chemokine secretion in comparison to standard immune activators such as lipopolysaccharide (LPS) and PMA/ionomycin. The NP treatment showed adjuvant-like activity with induction of inflammatory cytokines, growth factors, and select chemokines in peripheral blood mononuclear cells (PBMCs) of both young (n = 6) and older adults (n = 4), yet the degree of activation was generally less than LPS. Treatment with Mi or CDN resulted in minimal induction of cytokines and chemokine secretion with the exception of increased IFN-α and IL-12p70 by CDN. Age-related decreases were observed across multiple cytokines and chemokines, yet IFN-α, IL-12, and IL-7 production by NP or CDN stimulation was equal to or greater than in cells from younger adults. Consistent with these results in aged humans, a combination nanovaccine composed of NP, Mi, and CDN administered to aged mice resulted in a greater percentage of antigen-specific CD4^+^ T cells and greater effector memory cells in draining lymph nodes compared to an imiquimod-adjuvanted vaccine.

**Conclusions:**

Overall, our novel biomaterials demonstrated a modest induction of cytokine secretion with a minimal inflammatory profile. These findings suggest a unique role for biomaterial nanoadjuvants in the development of next generation vaccines for older adults.

**Supplementary Information:**

The online version contains supplementary material available at 10.1186/s12979-023-00349-5.

## Background

With current vaccines designed and optimized for younger adults, there is a need to provide efficacious vaccines for at-risk populations such as older adults (i.e., ≥ 65 years old). By 2050, it is expected that the population of older adults will double [1]; yet core vaccines recommended for older adults (including influenza virus, varicella zoster virus, pneumococcal, tetanus, and diphtheria vaccines) demonstrate age-dependent efficacy [1–3]. A study examining post-influenza virus vaccination responses found that more than half of the participating older adults failed to seroconvert to any of the vaccine strains [4]. This poor vaccine efficacy in older adults is often attributed to immune deficiencies (i.e., immunosenescence) that develop as an individual ages, resulting in decreased B and T cell proliferative capacity and function [1–3]. However, age-associated impairments to antigen presenting cells (APCs), which play a critical role in shaping adaptive B and T cell immune responses [5], may present a unique challenge and opportunity for new vaccination strategies.

One of the key characteristics of age-associated APC deficits is the imbalance of inflammation and immune activation (i.e., inflammaging). Baseline levels of proinflammatory cytokines such as IL-6 and TNFα have been found to be elevated in both aged human and aged murine cells [4, 6]. This dysregulated proinflammatory state has been associated with decreased phagocytic capacity, expression of pattern recognition receptors, and antigen presentation, and increased reactive oxygen species (ROS) [4, 6–10]. Additionally, while baseline levels of proinflammatory cytokines are raised overall, dendritic cells (DCs) from aged individuals may exhibit smaller increases in cytokine production upon stimulation [4].

Strategies to improve vaccine efficacy in older adults often include the addition of adjuvants; however, there must be careful selection of the adjuvants used in order to balance enhancing immune activation without exacerbating inflammaging [2]. To this end, novel adjuvants such as polyanhydride nanoparticles, pentablock copolymer micelles, and cyclic dinucleotides (CDN; a STING agonist), have been shown to provide a unique combination nanovaccine platform that may be suitable for older adults [11], yet few studies have comparatively evaluated effects of nanoadjuvants on cells from aged and young humans.

Polyanhydride nanoparticles (NP) are biodegradable, polymeric particles that sustain the release of antigenic payloads [12, 13]. These particles have been shown to promote activation of APCs [14], long-lived antibody responses [13, 15–17], and cell-mediated immunity [17–19]. Stimulation of dendritic cells from young and aged mice with NP upregulated co-stimulatory molecules CD40 and CD86 without overt proinflammatory cytokine production [11, 20]. Similarly, pentablock copolymer micelles (Mi) based on poly(diethylaminoethyl methacrylate) (PDEAEM) and Pluronics have also been shown to demonstrate rapid induction of antibody responses and enhanced antigen internalization, without induction of proinflammatory cytokines in young or aged DCs [11, 21]. Additionally, while CDN induces modest levels of ROS, their unique metabolic profile results in enhanced antibody titers in both young and aged mice [22]. Most notably, all three adjuvants (NP, Mi, and CDN) have been shown to preserve DC mitochondrial functionality without the induction of excessive ROS in mice [20–22]. When combined into a single dose influenza A virus nanovaccine formulation, this combination nanoadjuvant formulation induced protective immunity of aged mice against viral challenge [11]. In separate findings, NP combined with the TLR9 adjuvant CpG delivered by a mucosal route induced local T cell immunity as well as antibody responses resulting in enhanced protection from influenza viral challenge [17]. Of note, although this nanovaccine induced robust cellular immunity within the lungs, vaccinated mice demonstrated reduced pulmonary resistance (i.e., inflammation) [17], suggesting that such vaccines may be ideal for inducing immunity in older adults without exacerbating tissues more prone to inflammation. Together, these findings show that NP-based vaccines provide protection across immunization routes and in combination with different adjuvants.

While we have previously examined the effects of novel biomaterial nanoadjuvants in aged and young mice [11], in this work, we sought to examine the effect of NP, Mi, and CDN on cytokine and chemokine responses in peripheral blood mononuclear cells (PBMCs) from young and older human adults. A modest activation of cytokines without an overt inflammatory profile might suggest that these nanoadjuvants will show promise as vaccine delivery platforms for older adults [2]. Assuming the response of human cells to nanoadjuvants would parallel the response in mice, we expected that the cytokine profile elicited by nanoadjuvants would reflect activation of APCs rather than T cells [14]. Because these biomaterials had not yet been tested on cells from humans and their activation potential across immune cell types was unknown, cytokine responses induced by nanoadjuvants were compared to those induced by lipopolysaccharide (LPS; innate immunity) and PMA/ionomycin (PMA; T cell activation). Human PBMCs as a mixed cell population were examined rather than isolated cells to reflect the age-associated changes in the proportion of cell subset changes that may contribute to overall cytokine response. Another concern in the context of aging is dysregulation across multiple immune cell types, which may influence overall cytokine response [23], as investigators have shown that the presence of lymphocytes influences the age-related inflammatory profile of monocytes [24]. Therefore, it was of interest to use a mixed population of innate and adaptive immune cells. Herein we illustrate the ability of novel nanoadjuvants and CDN to alter cytokine and chemokine profile of human PBMCs in a unique manner that varies by age status. Based on these findings with human PBMCs, a vaccine formulation containing nanoadjuvants and CDN was tested in a mouse model to evaluate T cell responses downstream of APC priming.

## Results

### Human DC subsets are altered in aging

**Fig. 1:**
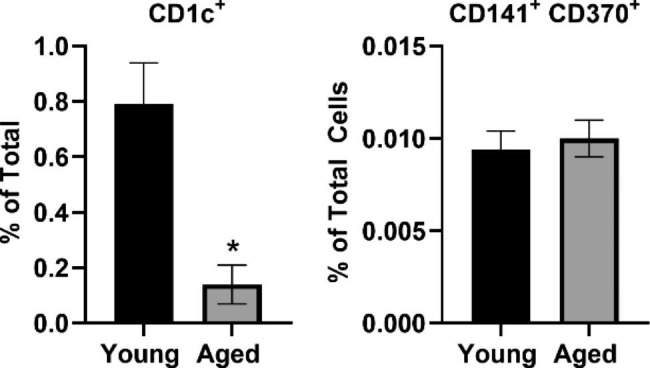
Percentage of conventional DC subsets in blood varies by age. Flow cytometry was used to identify DC subsets in blood samples obtained from young or aged adults. The CD1c^+^ DC subset (cDC2) as a percentage of total cells is reduced in aged adults compared to young (* indicates significant effect of age, *p* < 0.05). There was no significant difference by age for the CD41^+^ CD370^+^ DC subset (cDC1) as a percentage of total cells (n = 3 aged, n = 3 young). Error bars represent the standard error of the mean (SEM).

The impact of age on the relative composition of human peripheral blood DC subsets may influence overall response to vaccine components, and, therefore, initial studies evaluated the two primary conventional DC subsets, cDC1 and cDC2. Human peripheral blood was collected from young (n = 3) and aged (n = 3) adults (Table 1) and examined for conventional DC subsets. The frequency of CD1c^+^ cells (reflective of the cDC2 subset) as a percentage of total PBMCs was found to be reduced in older compared to younger adults (Fig. 1). However, there were no significant differences by age in the CD141^+^ CD370^+^ DCs (cDC1 subset), although the frequency of cDC1 was less than cDC2 cells, consistent with other literature [25].

**Table 1:** Characteristics of participants for the experiments to evaluate dendritic cell subsets

Demographic information- dendritic cell subsets	Young (mean ± s.e.m.)	Aged (mean ± s.e.m.)	
Age (years)	29.0 ± 2.0	71.3 ± 1.7*	*p < 0.001
Body weight	146.0 ± 12.8	155.3 ± 13.9	*p* = 0.648
Female	n = 2	n = 2	
Male	n = 1	n = 1	

### Stimulation with novel adjuvants alter cytokine and chemokine production by PBMCs

**Fig. 2:**
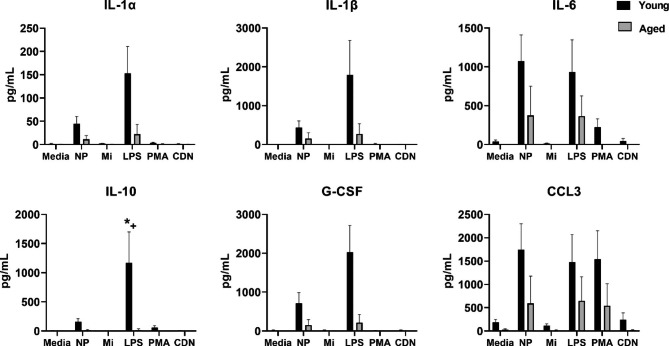
Cytokine or chemokine production by young or aged PBMCs is altered by NP and LPS treatment. PBMCs from young (n = 6) or aged (n = 4) adults were incubated with either no adjuvants (media), polyanhydride nanoparticles (NP), Micelles (Mi), LPS, PMA/ionomycin (PMA), or cyclic dinucleotide (CDN) adjuvant for 24 h and analyzed for production of cytokines, chemokines, or growth factors. The results showed that a significant main effect of treatment was observed for IL-1α, IL-1β, IL-6, IL-10, G-CSF and chemokine CCL3 (*p* < 0.05). A main effect of age was observed for IL-1α, IL-6, G-CSF, and CCL3 (*p* < 0.05), and a significant age by treatment interaction for IL-10 was found as indicated by *+ in the IL-10 graph (*p* < 0.05). Post-hoc analysis showed that LPS and NP induced greater IL-1α, IL-1 β, and IL-6 than media alone. LPS induced greater G-CSF and CCL3 than NP, and NP treatment resulted in greater G-CSF than media alone. Only LPS and PMA treatment resulted in greater CCL3 than media alone. For all significant age effects, cytokine concentration produced by young was greater than aged. The results for IL-10 showed an interaction between treatment and age, suggesting that the IL-10 response to LPS was greater in young compared to aged, as only LPS treatment was greater than media

Based on our previous work [14, 26] it was anticipated that biomaterials with different chemical and physical properties would alter the production of cytokines, but the response of human primary cells or the effect of aging on cytokine or chemokines induced by these biomaterials was unknown. Peripheral blood mononuclear cells from young (n = 6) and aged (n = 4) participants (Table 2) were incubated for 24 h with biomaterials NP, Mi, adjuvant CDN, or immune stimulators LPS or PMA/ionomycin. Stimulation with NP or LPS increased the secretion of a select set of inflammation-associated cytokines and chemokines (IL-1α, IL-1β, IL-6, IL-10, G-CSF, CCL3) from PBMCs obtained from both young and aged adults, and may reflect a pattern aligned with activation of innate immunity (Fig. 2). A two-way ANOVA was used to determine the effect of in vitro treatment, effect of age, or interaction. The results demonstrated that a significant main effect of treatment was observed for G-CSF, IL-6, IL-1β, IL-1α, IL-10, and chemokine CCL3. A main effect of age was observed for G-CSF, IL-6, IL-1α, CCL3, and a significant age by treatment interaction for IL-10 was found (Fig. 2). With respect to post-hoc analysis, LPS and NP induced greater IL-6, IL-1β, IL-1α than media alone. LPS induced greater G-CSF and CCL3 than NP, and NP treatment resulted in greater G-CSF than media alone. Only LPS and PMA treatment resulted in greater CCL3 than media alone. For all significant age effects, cytokine concentration produced by young was greater than aged. The results for IL-10 showed an interaction between treatment and age, suggesting that the IL-10 response to LPS was greater in young compared to aged, as only LPS treatment was greater than media. A visualization of the overall pattern of cytokine, chemokine, or growth factor response resulting from activation by LPS and NP is shown as a radial plot in Supplementary Fig. 1, with the general pattern suggesting that LPS is more stimulatory than NP.

**Table 2:** Characteristics of participants for the experiments designed to assess the effect of LPS, PMA-ionomycin, Nanoparticles, Micelles, or Cyclic Dinucleotide on cytokine and chemokine response

Demographic information- cytokine and chemokine	Young (mean ± s.e.m.)	Aged (mean ± s.e.m.)	
Age (years)	24.6 ± 1.7	78.5 ± 2.3*	*p < 0.001
Body weight	169 ± 17.0	163.7 ± 20.0	*p* = 0.821
Serum IL-6	12.9 ± 4.9	20.1 ± 2.3	*p* = 0.325
Female	n = 3	n = 1	
Male	n = 3	n = 3	

**Fig. 3:**
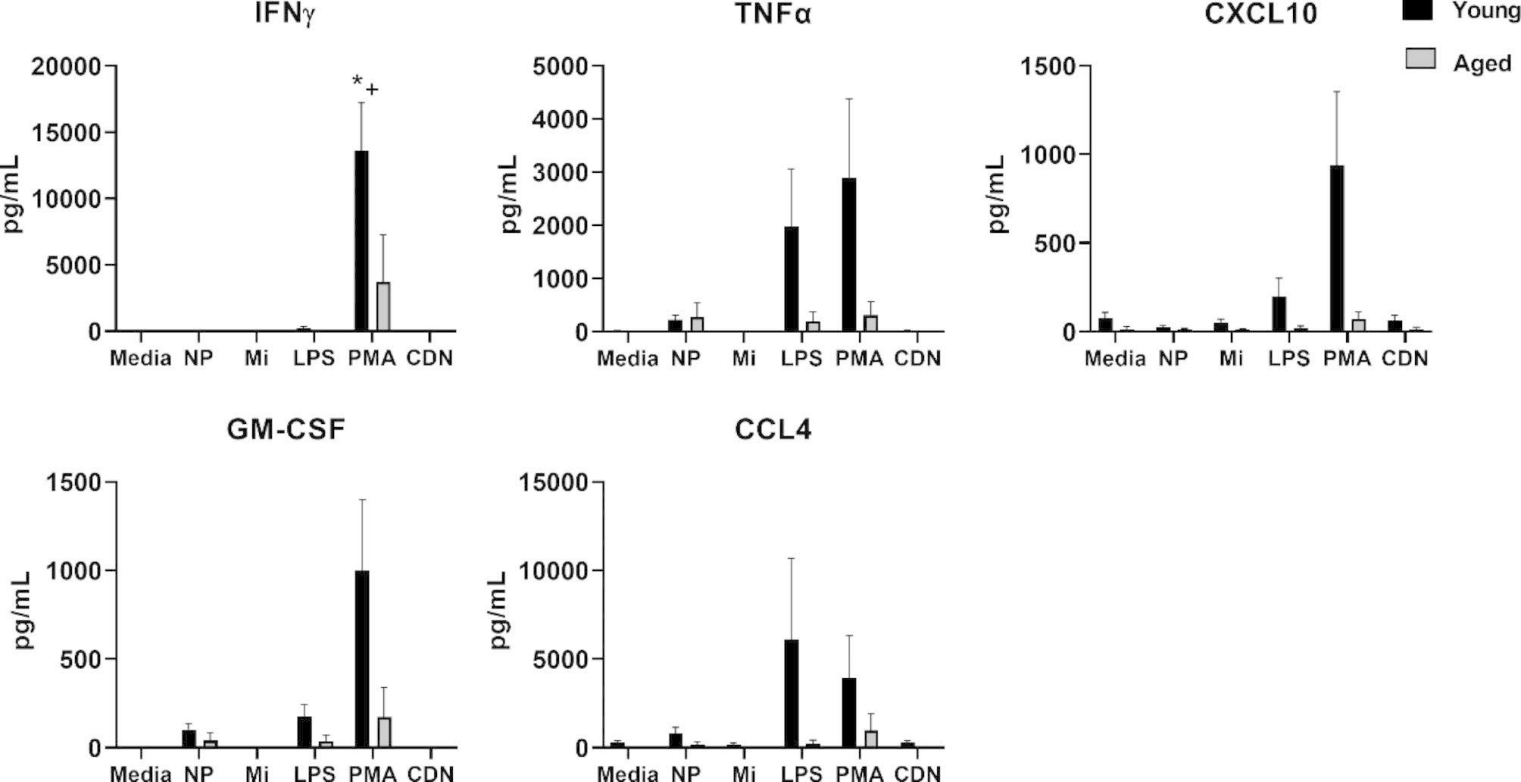
Cytokine or chemokine production by young or aged PBMCs is altered by PMA/ionomycin, LPS, or NP treatment. PBMCs from young (n = 6) or aged (n = 4) adults were incubated with either no adjuvants (media), polyanhydride nanoparticles (NP), Micelles (Mi), LPS, PMA/ionomycin (PMA), or cyclic dinucleotide (CDN) adjuvant for 24 h and analyzed for production of cytokines, chemokines, or growth factors. A main effect of treatment was found for IFN-γ, TNFα, CXCL10, GM-CSF (*p* < 0.05) and trend for CCL4. In all comparisons with significant treatment effects, post hoc analysis show that PMA was greater than media alone (*p* < 0.05). LPS induced greater production of IFN-γ, GM-CSF and CXCL10 compared to media alone, and post hoc analyses also showed that NP treatment was greater than media alone for GM-CSF only, with a trend (*p* = 0.06) for TNFα > media. A main effect of age was observed for all cytokines shown (*p* < 0.05), with lower concentrations in culture supernatants of cells obtained from older adults. A significant treatment by age interaction for IFN-γ activation was found as indicated by *+ in the IFN-γ group

A separate set of cytokines, chemokines, and growth factors were increased by treatment with PMA/ionomycin, along with some effects of LPS and a modest effect of NP (Fig. 3). A main effect of treatment was found for IFN-γ, TNFα, GM-CSF, CXCL10, and trend for CCL4, and in all comparisons with significant treatment effects, PMA was greater than media alone based on post-hoc analyses. LPS induced greater production of IFN-γ, GM-CSF and CXCL10 compared to media alone, and post hoc analyses also showed that NP treatment was greater than media alone for GM-CSF only, with a trend (*p* = 0.06) for TNFα > media. A main effect of age was observed for all cytokines shown, with lower concentrations in culture supernatants of cells obtained from older adults (Fig. 3). A significant treatment by age interaction for IFN-γ activation was found.

**Fig. 4:**
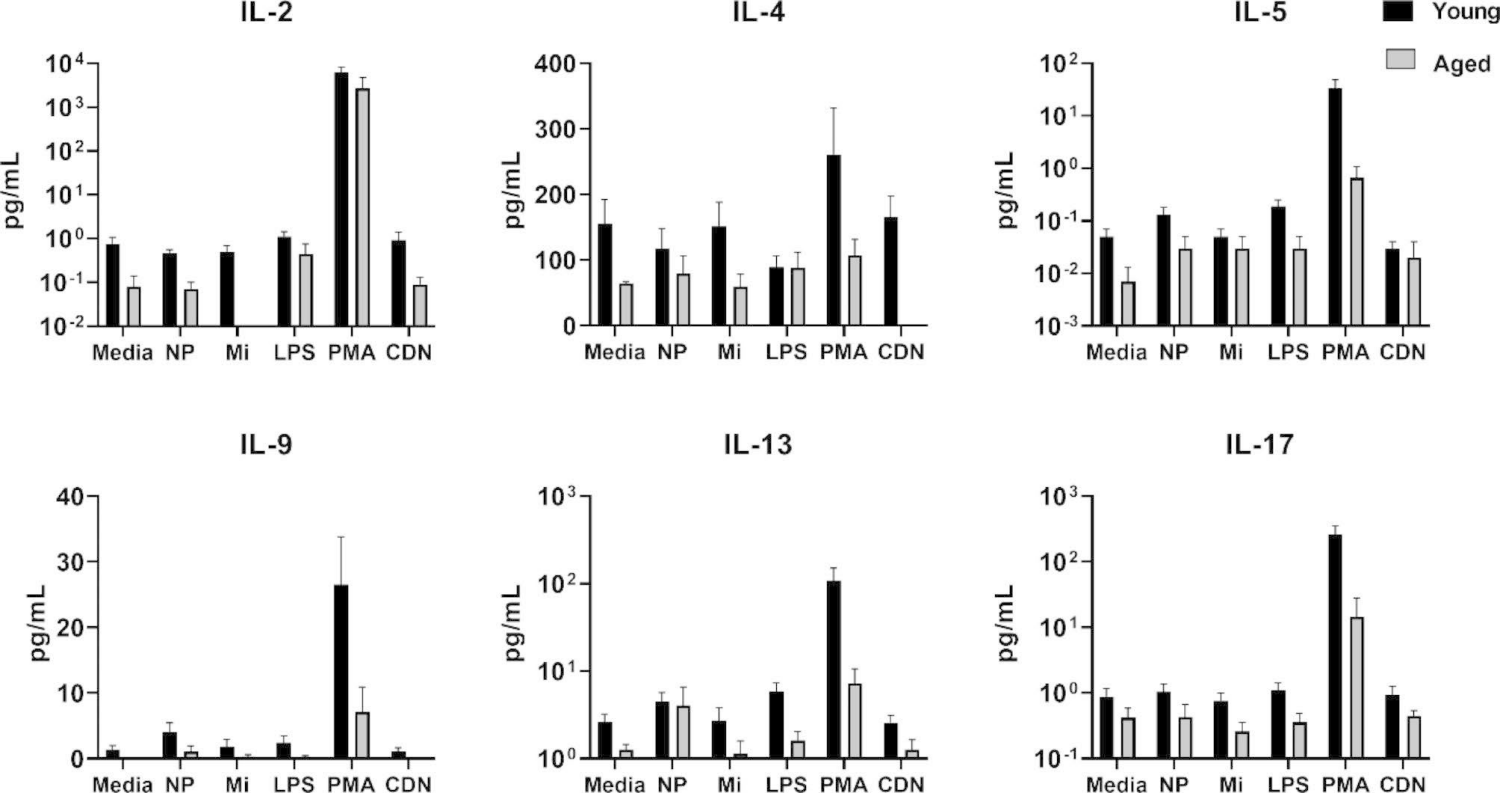
Cytokine production by young or aged PBMCs is altered by PMA/ionomycin treatment. PBMCs from young (n = 6) or aged (n = 4) adults were incubated with either no adjuvants (media), polyanhydride nanoparticles (NP), Micelles (Mi), LPS, PMA/ionomycin (PMA), or cyclic dinucleotide (CDN) adjuvant for 24 h and analyzed for production of cytokines typically associated with secretion by T cells (IL-2, IL-4, IL-5, IL-9, IL-13, IL-17). A main effect of treatment was found for all cytokines (*p* < 0.05), with significantly increased concentrations induced by PMA/ionomycin treatment compared to media alone or other treatments, as based on post-hoc analysis (except IL-4 as trend). NP increased IL-9 as compared to media alone (*p* < 0.05). Also, a main effect of age was found with greater levels in supernatants from young adults relative to older adults (*p* < 0.05). with the exception of IL-5 (*p* = 0.06)

A main effect of treatment was found for all cytokines typically associated with secretion by T cells (IL-2, IL-4, IL-5, IL-9, IL-13, IL-17; Fig. 4), with significantly increased concentrations induced by PMA/ionomycin treatment compared to media alone or other treatments, as based on post-hoc analysis (except IL-4 as trend). The only cytokine increased by a treatment other than PMA/ionomycin was IL-9, as NP increased IL-9 as compared to media alone. Also, a main effect of age was found with greater levels in supernatants from young adults relative to older adults with the exception of IL-5 (*p* = 0.06) (Fig. 4).

**Fig. 5:**
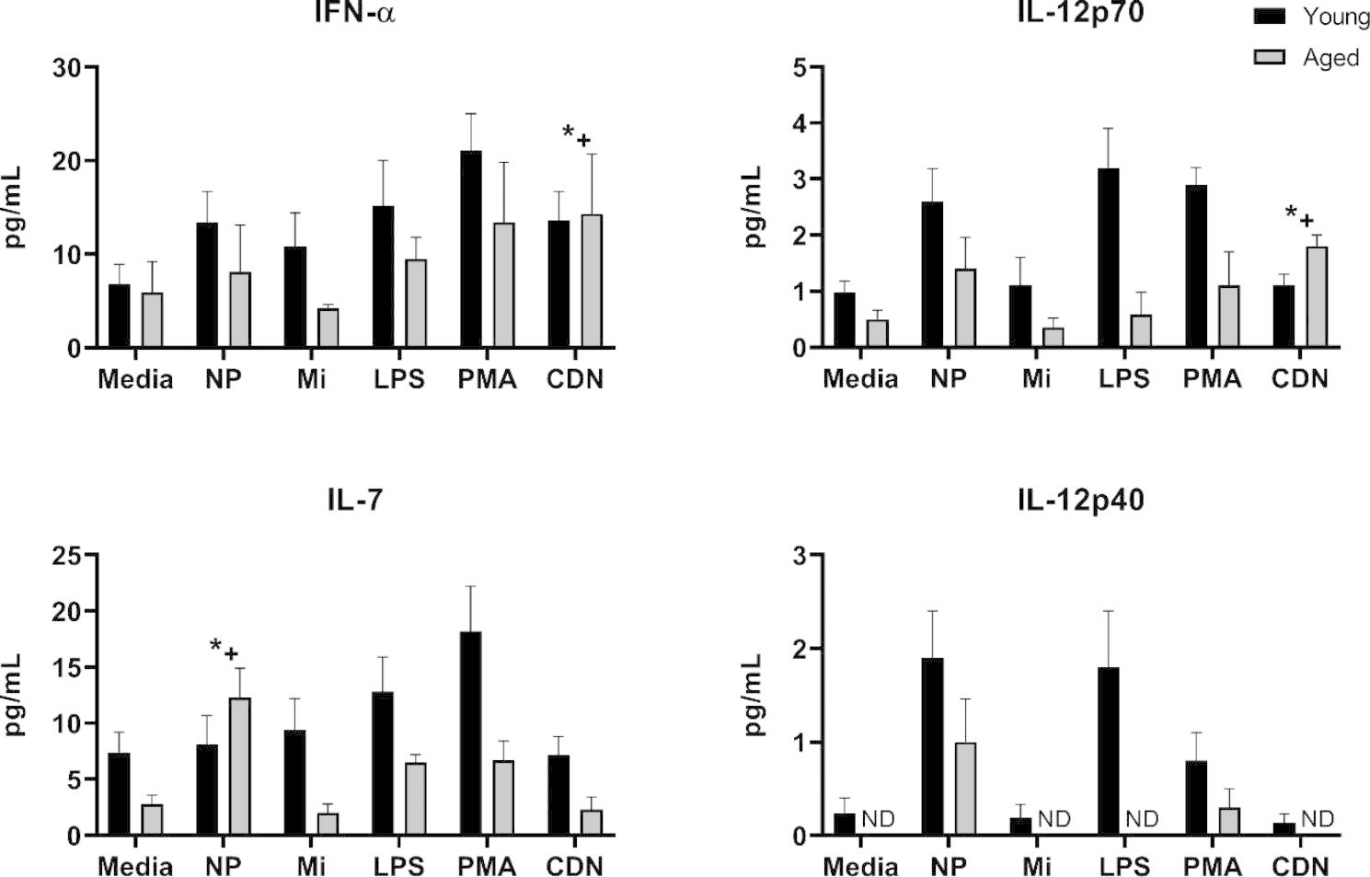
Cytokine production by young or aged PBMCs is differentially altered by NP, CDN, LPS or PMA/ionomycin treatment. PBMCs from young (n = 6) or aged (n = 4) adults were incubated with either no adjuvants (media), polyanhydride nanoparticles (NP), Micelles (Mi), LPS, PMA/ionomycin (PMA), or cyclic dinucleotide (CDN) adjuvant for 24 h and analyzed for production of cytokines. A significant main effect of treatment was found for IFN-α, IL-7, IL-12p40 and IL-12p70 (*p* < 0.05), a main effect of age for all cytokines (*p* < 0.05), and a treatment by age interaction for IL-12p70, IL-7 (*p* < 0.05); and for IFN-α, a trend to interaction (*p* = 0.06). Due to interaction effects, post-hoc analyses identified treatment differences by age. For IFN-α, only CDN treatment was greater than media in aged (*+, *p* < 0.05), but CDN did not significantly increase IFN-α in young. Treatment with PMA, LPS, and NP increased IFN-α in young only (p < 0.05). For IL-12p70, only CDN increased IL-12p70 in aged (*+, *p* < 0.05). In young PMA treatment increased IL-12p70 (*p* < 0.05), with a similar trend for LPS (*p* = 0.06) and NP (*p* = 0.09). IL-7 was increased by NP treatment in aged only (*+, *p* < 0.05), but IL-7 was increased in young by PMA (*p* < 0.05) and trend for LPS (*p* = 0.058)

Although the trend of lower cytokine production in aged relative to young was a common finding, for a small set of cytokines (IFN-α, IL-7, and IL-12p70) the age-related changes tended to vary by treatment. A significant main effect of treatment was found for IFN-α, IL-7, IL-12p40 and IL-12p70, a main effect of age for all cytokines, and a treatment by age interaction for IL-12p70, IL-7 (*p* < 0.05), and for IFN-α a trend to interaction (*p* = 0.06) (Fig. 5). In subsequent post-hoc analyses, the treatments for which young and aged responded differently were identified. In older adults, only CDN treatment increased IFN-α (indicated by *+, Fig. 5), whereas PMA/ionomycin, LPS, and NP significantly increased IFN-α in young adults. With respect to IL-12p70, the findings are similar to IFN-α in that only CDN increased IL-12p70 in cells from older adults (indicated by *+, Fig. 5) whereas PMA/ionomycin significantly increased IL-12p70 in young, with a similar trend for LPS (*p* = 0.06) and NP (*p* = 0.09). IL-7 concentration was increased only by NP treatment in aged (indicated by *+, Fig. 5). In contrast IL-7 was significantly increased by PMA/ionomycin in young with a trend towards an increase for LPS treatment (*p* = 0.058). IL-12p40 production was largely undetectable in supernatant collected from cells from older adults, with only NP inducing IL-12p40 relative to media alone in both aged and young (Fig. 5).

Table 3 shows the results of cytokines, chemokines, and growth factors that showed no main effect of treatment and did not align with the pattern of results shown in Figs. 2, 3 and 4. A significant main effect of age was found for CCL2, CCL7, CCL11, CCL22 and IL-8.

**Table 3:** Mean cytokine/chemokine concentration (pg/mL) ± standard error in Aged (65 + years of age) and Young (18–49 years of age) individuals. PBMCs were incubated with either media (control), LPS, polyanhydride nanoparticles (NP), pentablock copolymer micelles (Mi), PMA/ionomycin (PMA), or cyclic dinucleotides (CDN) to test the effects of vaccine adjuvants on cytokine/chemokine production. Significant effects of Treatment (Trt) Age, or Trt × Age interaction from generalized linear model (GLM) analysis are shown. * p ≤ 0.05, †p > 0.05 but showing distinct trend. Dashed lines signify no effect of Trt, Age, or significant interaction in cytokine/chemokine level. For treatments that reached statistical significance, post-hoc results are shown

	AGED							YOUNG							
	Control	LPS	NP	Mi	PMA	CDN		Control	LPS	NP	Mi	PMA	CDN		Sig. Effects from GLM
**CD40L**	22.93 ± 7.39	17.59 ± 6.64	49.1 ± 34.28	42.23 ± 29.49	64.87 ± 46.26	21.05 ± 10.62		17.62 ± 8.46	17.53 ± 7.46	22.55 ± 11.36	19.25 ± 8.85	44.55 ± 14.04	16.28 ± 5.1		--
**EGF**	22.79 ± 6.17	21.95 ± 7.06	28.88 ± 4.85	24.53 ± 7.88	24.47 ± 8.03	22.27 ± 10.45		24.71 ± 2.95	28.55 ± 3.43	27.6 ± 2.77	24.64 ± 4.07	28.48 ± 3.94	26.29 ± 1.94		--
**CCL11**	6.45 ± 1.73	8.84 ± 2.94	11.75 ± 2.07	7.24 ± 1.61	10.55 ± 1.34	8.6 ± 1.4		14.12 ± 2.55	13.92 ± 2.83	16.91 ± 2.06	12.91 ± 2.74	16.15 ± 2.91	16.68 ± 0.88		Age *
**Flt3L**	0 ± 0	0.61 ± 0.61	0 ± 0	0.53 ± 0.53	0.7 ± 0.7	0 ± 0		1.44 ± 0.66	1.71 ± 0.64	1.78 ± 0.76	1.34 ± 0.73	2.64 ± 0.87	1.84 ± 0.63		--
**CX3CL1**	22.93 ± 7.39	15.68 ± 0.74	30.57 ± 11.96	16.01 ± 3.07	22.82 ± 5.73	15.95 ± 5.65		15.96 ± 2.33	24.38 ± 4.16	20.92 ± 3.76	20.72 ± 5.58	34.07 ± 5.93	19.29 ± 4.3		--
**IL-1Ra**	10.17 ± 8.59	13.01 ± 11.52	25.63 ± 20.75	4.96 ± 3.27	8.29 ± 5.06	8.29 ± 5.94		122.79 ± 56.27	172.77 ± 71.95	88.98 ± 42.1	95.1 ± 38.41	62.09 ± 22.09	104.37 ± 35.73		--
**IL-3**	0.79 ± 0.07	0.68 ± 0.08	1.02 ± 0.22	0.91 ± 0.2	4.23 ± 3.21	0.85 ± 0.07		0.82 ± 0.09	0.75 ± 0.09	0.95 ± 0.08	0.95 ± 0.13	64.77 ± 29.53	0.77 ± 0.11		Trt * PMA > media
**IL-8**	476.55 ± 438.1	1203.36 ± 1069.23	1273.92 ± 1255.38	203.16 ± 175.43	770.29 ± 651.71	313.76 ± 269.14		4851.7 ± 1632.21	3884.75 ± 1208.16	3363.05 ± 1097.72	3675.12 ± 1244.26	3570.11 ± 1166.03	4063.73 ± 1311.12		Age *
**CCL2**	605.93 ± 533.43	53.46 ± 39.91	420.6 ± 401.74	207.93 ± 166.66	105.99 ± 89.67	321.89 ± 244.61		2833.08 ± 880.65	1013.49 ± 537.84	2091.94 ± 904.24	2612.05 ± 823.35	1796.08 ± 735.31	2688.71 ± 855.83		Age *
**CCL7**	47.78 ± 41.39	1.74 ± 1.14	4.4 ± 3.41	21.91 ± 18.35	33.12 ± 30.27	36.77 ± 29.4		1364.98 ± 738.76	21.28 ± 8.61	23.72 ± 10.64	558.32 ± 228.69	243.31 ± 84.6	822.96 ± 402.48		Age *
**CCL22**	19.52 ± 3.57	24.33 ± 11.13	11.41 ± 5	17.51 ± 4.07	37.03 ± 15.05	12.88 ± 3.55		250.16 ± 107.1	152.63 ± 47.81	20.48 ± 5.34	191.65 ± 76.4	214.41 ± 86.02	271 ± 130.35		Age *
**PGDF**	66 ± 23.08	54.95 ± 20.49	72.23 ± 37.4	76.5 ± 29.64	106.56 ± 49.73	67.24 ± 32.34		57.42 ± 15.05	54.97 ± 14.11	39.6 ± 12.84	59.69 ± 16.75	81.13 ± 21.61	53.02 ± 10.13		--
**CCL5**	5039.57 ± 1003.87	6921.69 ± 2813.84	4102.32 ± 2079.83	5491.54 ± 1862.46	6944 ± 2721.11	4209.14 ± 1729.56		5474.51 ± 1262.09	5160.44 ± 594.57	2165.86 ± 782.96	5156.84 ± 1328.17	5769.67 ± 725.3	3331.47 ± 719.67		Trt * NP<,media
**TGF-**α	0.78 ± 0.02	0.78 ± 0.06	0.79 ± 0.09	0.74 ± 0.05	0.73 ± 0.04	0.81 ± 0.07		1.07 ± 0.18	1.72 ± 0.38	0.93 ± 0.09	3.13 ± 2.26	0.92 ± 0.06	0.99 ± 0.14		--
**TNF-β**	0.08 ± 0.08	0.24 ± 0.1	0.2 ± 0.15	0.14 ± 0.09	0.99 ± 0.73	0.27 ± 0.14		0.54 ± 0.19	0.59 ± 0.16	0.82 ± 0.27	0.56 ± 0.2	7.13 ± 2.36	0.62 ± 0.17		--
**VEGF**	7.77 ± 6.8	12.23 ± 5.53	16.38 ± 9.52	7.24 ± 6.28	22.75 ± 8.28	4.44 ± 2.31		29.76 ± 9.77	44.37 ± 11.36	40.34 ± 13.08	32.14 ± 11.02	43.55 ± 5.13	28.78 ± 9.11		Age†

### Combination nanovaccine enhanced in vivo CD4^+^ T cell responses in aged mice

**Fig. 6:**
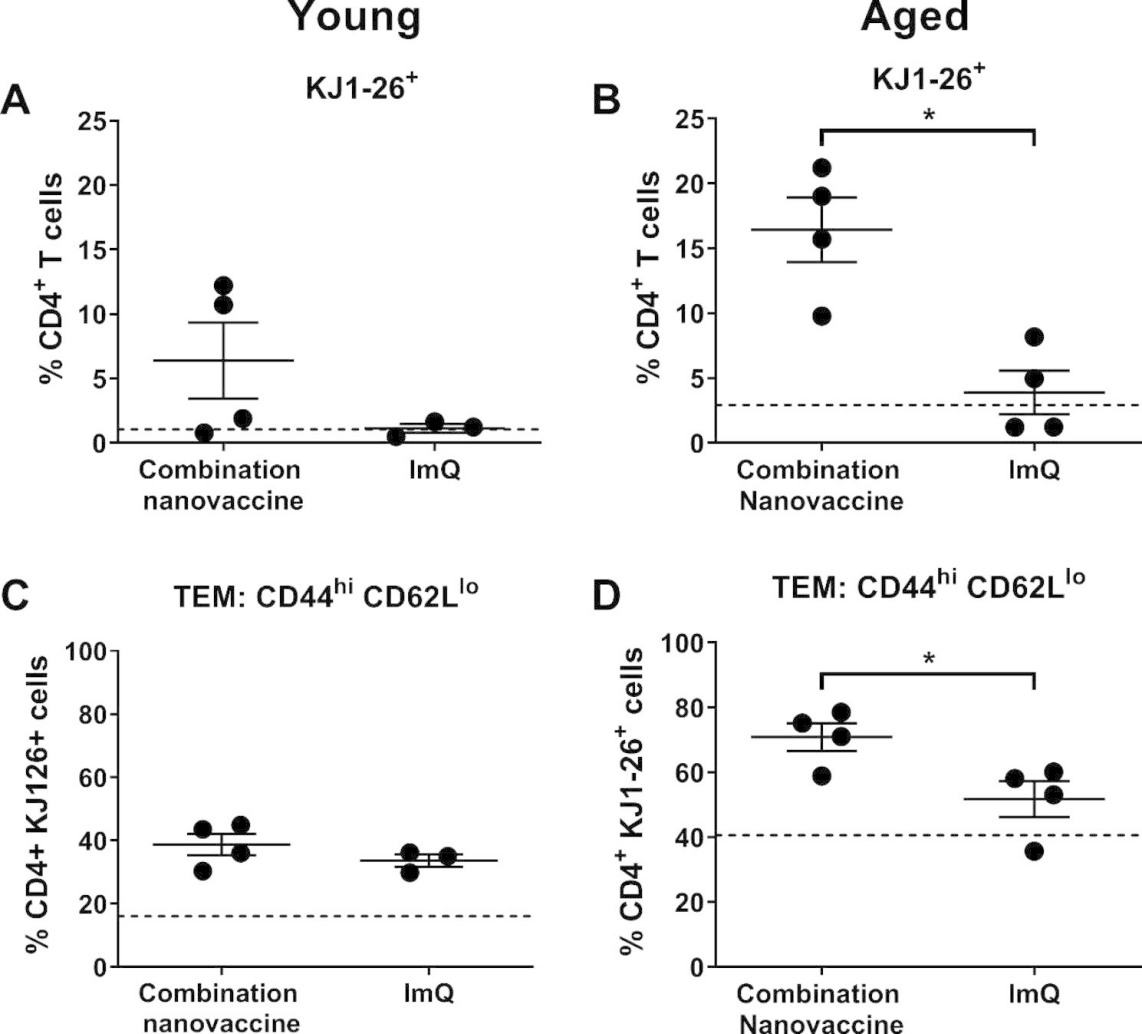
Combination nanovaccine enhanced antigen-specific CD4^+^ T cell responses in aged mice. Lymphocytes from DO11.10 Tg mice (0.5 × 10^6^ CD4^+^ KJ1-26^+^ cells) were adoptively transferred into young (2–6 mo) and aged (18–20 mo) BALB/c mice (n = 4) which were subsequently immunized with 100 µg of OVA adjuvanted with a combination nanovaccine (NP, Mi, and CDN) or 100 µg of OVA adjuvanted with imiquimod (ImQ). Percentage of antigen-specific CD4^+^ T cells (CD4^+^ KJ1-26^+^) or T-effector memory cells (TEM: CD4^+^ KJ1-26^+^ CD44^+^ CD62L^lo^) in the draining lymph nodes were examined at 3 days post-immunization. * Indicates significant difference between treatment groups, *p* ≤ 0.05. Error bars represent SEM. Dashed line represents background levels of the cell percentages in lymph nodes of mice treated with saline

As NP and CDN demonstrated several enhanced cytokine responses specific to PBMCs from aged adults (IL-12, IFN-α, IL-7) and these cytokines are considered to have a role in APC priming of T cell responses or T cell memory, an aged mouse vaccination model was used to test whether incorporating NP and CDN (along with relatively inert Mi as antigen carrier) would translate to downstream activation of T cells. In addition to their role in conferring “help” for antibody responses, effector CD4^+^ T cells (T helper cells) have been reported to be essential for mounting an effective memory CTL response during a secondary infection [27–29]. To assess the induction of antigen-specific (Ag-specific) CD4^+^ T cells in vivo following immunization, we utilized an adoptive transfer model with DO11.10 Tg mice. These mice are of the BALB/c background and carry T cell receptors (TCRs) that react to ovalbumin antigen (KJ1-26^+^). After adoptive transfer of DO11.10 T cells to aged wild-type (WT) BALB/c mice, animals were immunized with a combination nanovaccine (NP, Mi, and CDN) similar to formulations successfully used to enhance protection against influenza A virus in aged animals [11]. The response to this combination nanovaccine was compared to the response of a vaccine containing another adjuvant (imiquimod; ImQ) which has undergone testing in human trials to optimize vaccine responses in aged adults [30]. Immunization with the combination nanovaccine led to the greatest expansion in Ag-specific CD4^+^ T cells and T-effector memory (TEM) cells (CD44^hi^, CD62^lo^) in aged mice lymph nodes on day 3 post-immunization (Fig. 6). In contrast, immunization with the combination nanovaccine or imiquimod did not demonstrate expansion of antigen-specific or T-effector memory cells from young mice. Our results indicate that passively administered T cells effectively proliferated in the context of an “aged antigen presenting environment” when stimulated by combination nanoadjuvants (Fig. 6).

## Discussion

We identified the effect of biomaterials with adjuvant potential (NP and Mi) and the STING ligand adjuvant CDN in comparison with a commonly used PRR (LPS) or inducer of T cell activation (PMA) on cytokine and chemokine response of PBMCs obtained from young or aged adults. Our findings show that in general, cells from older adults respond to adjuvants and immune stimulators in a similar manner to young adults. However, aging may reduce the overall concentration of certain cytokines and chemokines or impact the degree to which a given treatment increases cytokine activation. Yet perhaps most relevant to vaccine design for older adults, a select subset of cytokines associated with APC activation and priming of T cells (IL-12p70, IFN-α) or T cell memory (IL-7) were activated by either NP or CDN to a similar or greater extent in aged relative to young (Fig. 5). A major limitation of these findings is the small number of participants that were included in the studies. Due to this limitation, these findings are considered preliminary. As these were the first experiments to evaluate the effect of the selected biomaterials on human primary cells, cells from younger and older adults were included given the emphasis on improving vaccines for underserved populations including older adults. However, additional studies are warranted with a larger number of participants to understand the variability in response based on age or other health conditions. The age-related effects of cytokine response to commonly used activating agents such as LPS observed in our studies is consistent with other findings [31], but future studies that include participants with a range of health conditions and across the aging spectrum are needed to identify the potential influence of host factors on immune response to adjuvants and biomaterials. It is possible that changes of immune cell subsets with aging could influence the cytokine and chemokine pattern, and we detected differences of DC subsets due to age. However, limitations of our findings include the small sample size and the use of freshly obtained cells for DC experiments whereas frozen cells were used in the cytokine experiments. Our findings suggest that a potential impact of age-related changes in cell subsets should be pursued in larger studies with the capacity to identify vaccine-formulation differences in cell subsets on a wide scale, as recently shown using mass cytometry CyTOF (Cytometry by Time-of-Flight) [32].

Given that it is challenging to screen adjuvants and evaluate subsequent T cell responses in humans, we used an aged mouse model to determine whether a combination of the adjuvants that showed promise in PBMCs from older adults (NP and CDN) would show evidence of T cell activation after vaccination. We also included Mi in the vaccine for their role in antigen delivery without activation of inflammatory cytokines (based on our findings of human PBMCs presented here and previous studies with APCs from mice [21]). The findings from the mouse immunization experiment showed evidence of T cell activation, as we observed a greater frequency of functional antigen-specific CD4^+^ T cells and CD4^+^ effector memory cells (CD44^hi^ CD62L^lo^) in the draining lymph nodes compared to a vaccine formulation containing the adjuvant ImQ + OVA (Fig. 6). ImQ was selected as the adjuvant in the comparison vaccine formulation because it has been tested in clinical trials for older adults [30]. Taken together, the findings from the human PBMC adjuvant experiments and the mouse immunization trial suggest that a careful screening and selection of adjuvants and antigen-delivery biomaterials with adjuvant properties that exhibit equivalent or greater efficacy among aged populations is a useful approach in optimizing vaccines for older adults. The in vitro assays of human PBMCs coupled with companion mouse immunization experiments may be useful pre-clinical approaches to identify more effective vaccines for older adults.

The ideal vaccine formulation for older adults may require “fine tuning” to find a balance between appropriate immune activation and overt inflammatory responses because inflammaging contributes to reduced vaccine efficacy [2, 33]. We examined the inflammatory potential of nanoadjuvants and the STING ligand CDN and observed that CDN did not induce secretion of inflammatory cytokines (IL-1β, IL-6, TNFα) but was effective in stimulating cytokines associated with APC activation and T cell priming. In contrast to CDN, we observed that NP significantly increased the production of cytokines or chemokines with inflammatory potential in cells from young and aged adults (IL-1α, IL-1β, IL-6, G-CSF, GM-CSF) (Figs. 2 and 3, Fig. S1). However, the overall degree of activation by NP was more modest as compared to LPS or PMA/ionomycin (Fig S1). The results showed that Mi are relatively inert immunologically with respect to the induction of cytokines and chemokines, and likely do not contribute directly to inflammatory activity in a vaccine formulation. NP and Mi have been shown to act synergistically to provide other benefits in vaccine formulations including sustained release, enhanced antigenic stability, and long-lived antibody responses [11, 12, 21, 34, 35]. Although the mouse experiments showed that the combination nanovaccine (adjuvanted with NP, Mi, and CDN) resulted in greater CD4^+^ T cell activation than ImQ (Fig. 6), further studies are needed to confirm that the mechanism underlying the improved T cell response involves induction of a differential inflammatory profile elicited by the vaccine. Although the findings of our studies are preliminary in terms of optimizing vaccine formulation for older adults, our results provide support for a combination nanovaccine formulation without potent inflammatory properties which may, in turn, be more efficacious for older adults.

Interestingly, NP and Mi both exhibited a minimal direct effect on the production of T cell-associated cytokines from human PBMCs, such as IFN-γ, IL-2, IL-4, IL-5, IL-9, IL-13, and IL-17 (Figs. 3 and 4). Similar to murine models, the effect of NP and Mi on T cell associated cytokines was minimal [14]. We observed treatment-specific age-related decreases across cytokines and chemokines. These findings are generally consistent with other literature showing reduced IL-12p70, TNFα, IL-6, IL-12p40 in DCs from older adults in response to TLR ligands [36], or in monocytes a decreased production of type I IFN (IFN-α), IL-1β, IFN-γ, and other chemokines not measured in our study, CCL20 and CCL8 [37]. We noted age-associated differences in the relative percentage of conventional DC populations and specifically a reduction in cDC2 cells (Fig. 1). It is possible that alterations in DC subsets can impact the cytokine profile in response to adjuvants; thus, further studies are warranted to tease out the impact of age on DC subset responses to vaccine adjuvants. Although we did observe age-associated decreases in cytokine response, we also identified several APC-related cytokines that responded equally well to NP or CDN in cells from aged and young adults. Furthermore, the combination vaccine formulation containing NP, Mi, and CDN showed evidence of enhanced CD4^+^ T cell responses in the draining lymph nodes of aged mice. Further exploration of mechanisms including downstream cell signaling pathways and APC-induced T cell activation could advance this line of research.

## Conclusions

Novel nanoadjuvants such as NP and CDN, but not Mi, were able to increase cytokine and chemokine production in PBMCs from older adults without an overt inflammatory profile. Using passive immunization of transgenic T cells, a nanovaccine comprising a combination of the nanoadjuvants was shown to enhance T cell responses in aged mouse immune environment. Overall, these findings are promising for the development of age-specific vaccine formulations with nanoadjuvants in combination with PRR ligands that exhibit low to moderate inflammatory profiles, but require additional testing with a larger n across a range of ages and health conditions.

## Methods

### Human participants

A total of 21 individuals were recruited to participate in this study. Participants were classified as either “aged” (aged 65+, n = 9) or “young” (aged 18–49, n = 12) adults. Of these participants, cells collected from several participants had poor viability or insufficient numbers to apply all in vitro treatments and were therefore not used in experiments. Therefore, studies to assess dendritic cell subsets included 6 individuals (3 young and 3 older adults, Table 1), whereas the experiments to evaluate the effect of in vitro treatment with biomaterial, adjuvants or immune activators included 10 individuals (n = 6 young, n = 4 older adults, Table 2). At the initial study visit, a health questionnaire was completed to determine eligibility for participation. Individuals with an immune disorder, cancer, or undergoing treatment with medication known to significantly alter immune response were excluded. If participants were unsure whether a condition or medication might be an exclusion criterion, a list of common conditions or medications was provided for the participant to examine, and clarification by the researcher was provided as needed. Categories of medications that would be expected to interfere substantially with immune response and/or immunometabolism include glucocorticoids (e.g., prednisone, dexamethasone), mTOR inhibitors (e.g., everolimus, sirolimus), calcineurin inhibitors (e.g., cyclosporin A, tacrolimus), antimetabolites (e.g., methotrexate), cytokine modulators (e.g., anakinra, rituximab), TNFα antagonists (e.g., etanercept), and nucleotide synthase inhibitors (e.g., azathriprine, leflunomide). This list provided examples of medications and categories that warrant exclusion but was not intended to be an all-encompassing list. Metformin warranted exclusion, not as an immunosuppressive medication, but one that could significantly alter immunometabolic responses. Statins, due to their widespread use and the mixed or modest findings with response to immunomodulatory properties did not warrant exclusion. Similarly, over the counter low dose aspirin therapy and NSAID usage did warrant exclusion. In Table 2, serum IL-6 is reported for the participants in which PBMC cytokines or chemokines were measured, but this information was not available for persons in which dendritic cell subsets were measured (Table 1). IL-6 can be considered a biomarker for underlying chronic disease, but serum IL-6 values were not used as inclusion/exclusion criteria. All procedures involving human subjects were approved by the Institutional Review Board at Iowa State University.

### Blood collection and isolation of PBMCs

Blood was collected from an antecubital vein into vacutainers containing the anticoagulant ethylenediaminetetraacetic acid (EDTA; for whole blood flow cytometry). An additional blood sample for isolation of PBMCs was collected in vacutainers containing sodium heparin. PBMCs were isolated via centrifugation of PBS-diluted whole blood (1:1 PBS:blood) over a Ficoll^®^Paque Plus density gradient (Sigma-Aldrich Inc., St. Louis, MO) per manufacturer’s instructions. Cells were adjusted to 1 × 10^7^ cells/mL and frozen in RPMI-1640 medium, with 30% fetal bovine serum (FBS) and 12% dimethyl sulfoxide (DMSO). Cells were stored in liquid nitrogen until subsequent use in PBMC cultures.

### Whole blood Flow Cytometry

**Fig. 7:**
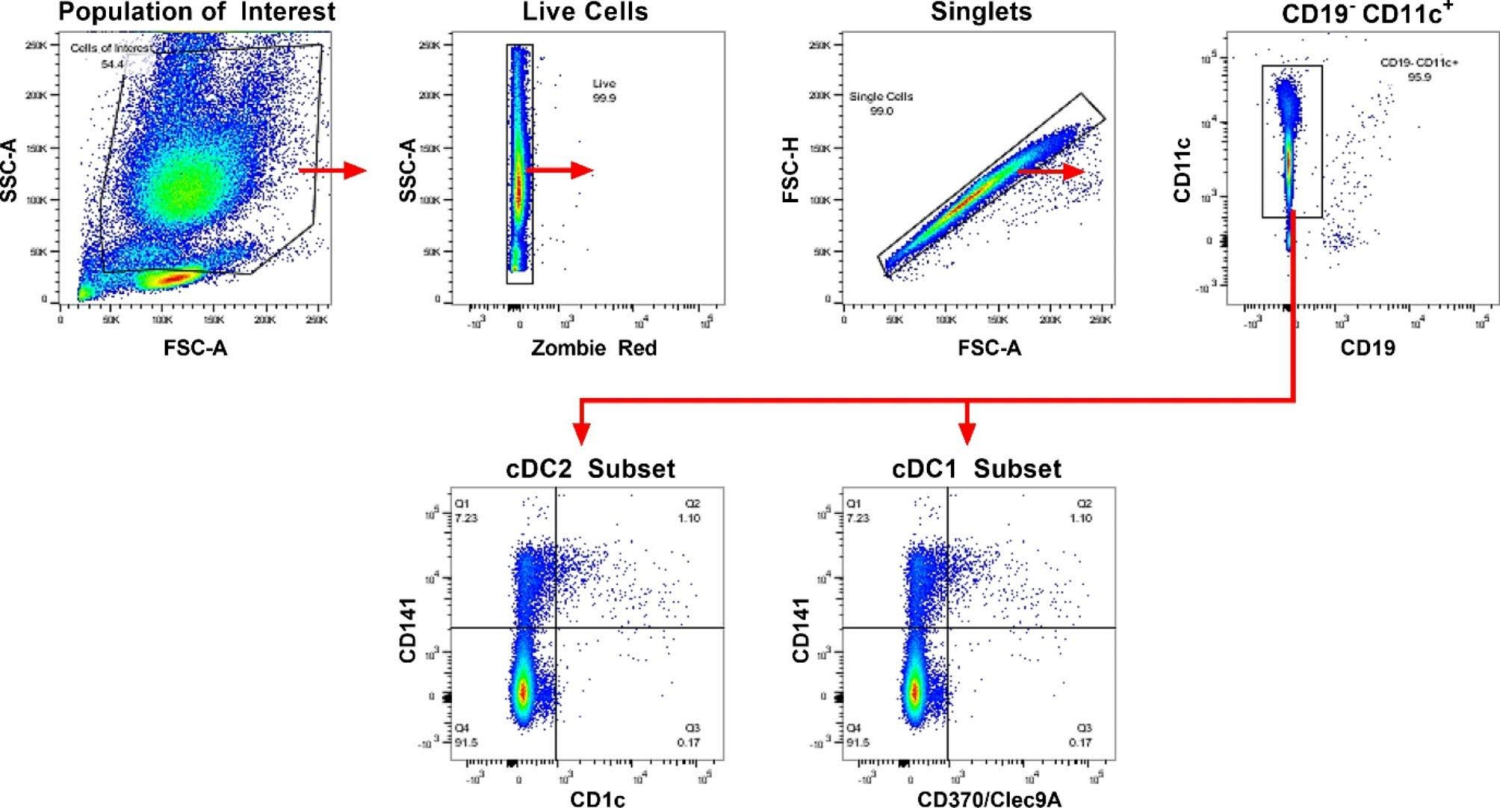
Gating scheme for human DCs. Whole blood was incubated with fluorochrome-conjugated antibodies as described in methods and gating strategy to identify conventional DCs as cDC1 (CD141^+^ CD370^+^) or cDC2 (CD1c^+^) is shown

To assess the impact of age on the frequency of DC populations, whole blood was analyzed via flow cytometry. Whole blood (100 µL) was incubated with fluorochrome-conjugated monoclonal rat or mouse anti-human antibodies for 30 min at 37 °C. Antibodies included: PE-Cy7 anti-CD141, APC-Cy9 anti-CD19, APC anti-CD370 (Clec9a), BV711 anti-CD11c, and BV421 anti-CD1c. Following incubation, red blood cells were lysed, the remaining cells washed with PBS, and incubated with 0.1 µL Zombie Red (BioLegend, San Diego, CA) in 100 µL PBS for 15 min. The cells were washed twice and resuspended in staining buffer containing bovine serum albumin. Cells were analyzed on a FACSCanto flow cytometer (BD Biosciences, Ashland, OR). Data were analyzed using FlowJo 10.0.7 software (BD Biosciences). Fluorescence minus one controls were used to set gates for all fluorescent parameters. Cells were gated based on forward and side scatter followed by exclusion of dead cells, debris, and doublets (Fig. 7). An initial gate for CD11^+^ CD19^-^ cells was followed by a quadrant to separate the CD141^+^ and CD1c^+^ cell subsets. The CD11c^+^ CD141^+^ CD1c^-^ gate was used to identify CD370^+^ (Clec9a) cells, a marker for the conventional DC type 1 (cDC1 subset). The number and percentages of the cDC1 subset and the CD1c^+^ subset (associated with the cDC2 subset) were determined for both aged and young samples.

### Biomaterial synthesis

Polyanhydride nanoparticles were produced as previously described [11, 13]. Briefly, a 20:80 molar ratio of 1,8-bis(*p*-carboxyphenoxy)-3,6-dioxaoctane (CPTEG) and 1,6-bis(*p*-carboxyphenoxy)hexane (CPH) diacids were polymerized via melt polycondensation [38, 39]. The resulting polymer was characterized by ^1^ H-nuclear magnetic resonance spectroscopy (^1^ H-NMR; Bruker DXR 500, Billerica, MA) to ensure purity and appropriate molecular weight (5,200 g/mol). Particles (empty or encapsulating 2 wt% ovalbumin (OVA)) were synthesized by dissolving the polymer and payload using 20 mg 20:80 CPTEG:CPH per mL of methylene chloride. The solution was sonicated for 30 s to ensure uniform distribution of the proteins. The solution was then precipitated into chilled pentane (-10 °C; 1:250 methylene chloride:pentane) and the resulting particles were collected by vacuum filtration. NP size and morphology were confirmed with scanning electron microscopy (FEI Quanta 250, FEI, Hillsboro, OR).

Pentablock copolymer based on DEAEM and Pluronic^®^ F127 were synthesized by atom transfer radical polymerization process as previously reported [40]. The purity and molecular weight (14,600 g/mol) of the resulting polymer was determined using ^1^ H-NMR (Bruker DXR 500). For the Mi formulation, a stock solution of 100 mg/mL of total polymer concentration was prepared with 41 wt% pentablock copolymer and 59 wt% Pluronic^®^ F127. This stock solution was diluted to the desired concentrations as detailed below.

### Adjuvant effect on PBMC cytokines, chemokines, and growth factors

In preparation for incubation of cells with adjuvants, PBMCs were rapidly thawed in a 37 °C water bath and washed twice. Cell viability was assessed and then cells were plated in 96-well round bottom plates at 5 × 10^5^ cells per well in a 200 µL volume. Cells were cultured in RPMI-1640 supplemented with 1% glutamine, 1% penicillin/streptomycin, 1% HEPES, 1% sodium pyruvate and 10% FBS. Blank (i.e., empty) NP were added to cells at 100 µg/well. Mi were added to cells at 0.015 µg/mL The concentration of NP and Mi was determined based on separate cell viability assays. Separate wells were treated with LPS from *Escherichia coli* O55:B5 (L6529, Sigma-Aldrich, St Louis, MO) at 0.1 µg/ml, CDN (cyclic diguanylate monophosphate; InvivoGen, San Diego, CA) at 0.5 µg/ml, cell stimulation cocktail (eBioscience, San Diego, CA) at a 1:500 dilution of the cocktail containing 40.5 µM Phorbol 12-Myristate 13-Acetate and 670 µM ionomycin, or cell culture medium alone. Cells were incubated at 37 °C at 5% CO_2_. Supernatants were collected and stored at -20 °C until analyzed. A multiplex kit (MILLIPLEX ® Human 41-plex cytokine/chemokine magnetic bead panel) was used to assess the concentration of cytokines and chemokines in the supernatants. Samples were analyzed using a Bio-Plex 200 system (Bio-Rad Laboratories Inc., Hercules, CA).

### Mice

Young Tg (DO11.10)10Dlo/J transgenic mice were purchased from Jackson Laboratories (Bar Harbor, ME). Female BALB/c mice were purchased from Charles River Laboratories (Wilmington, MA). Aged mice were approximately 18–20 months at the time of immunization. All mice were housed under specific pathogen-free conditions with all bedding, caging, water, and feed sterilized prior to use. All animal procedures were conducted with the approval of the Iowa State University Institutional Animal Care and Use Committee.

### Adoptive transfer of antigen-specific CD4 + T cells

Lymph nodes and spleens were isolated from DO11.10 Tg mice to make a homogenous cell suspension. The cells were stained with anti-KJ1-26 (a DO11.10 TCR antibody) and anti-CD4 antibodies and analyzed with flow cytometry before the adoptive transfer via tail-vein to track the number of antigen-specific CD4^+^ T cells being transferred to wild type (WT) BALB/c mice. Approximately half a million CD4^+^ KJ1-26^+^ cells were transferred to each recipient WT mouse. Aged recipient mice (n = 4–5/group) were subcutaneously immunized with 100 µg of OVA adjuvanted with 500 µg of NP, 5 mg Mi, and 20 µg CDN or 100 µg of OVA adjuvanted with 20 µg of imiquimod (InvivoGen). Cells from draining lymph nodes were harvested three days post-immunization and subsequently labeled with Zombie Aqua to test for viability, surface stained with anti-CD4 PerCP-Cy5.5, anti-KJ1-26-AlexaFluor647, anti-CD44 AlexaFluor700, anti-CD62L APC-eFlour780.

### Statistical analyses

The experiments involving human cells were analyzed using IBM SPSS Statistics version 25.0 (IBM Corp., White Plains NY). A general linear model approach was used including factors of age and in vitro treatment condition (two-way ANOVA, with Sidak post-hoc multiple comparison). As variability in cytokine response with a small number of human participants was expected, a Shapiro-Wilk test for normality was evaluated for each cytokine and chemokine, and data was log transformed for cytokines and chemokines that did not meet the test for normality. Although mean values of cytokines, chemokines and growth factors (pg/mL) are shown in figures or Table 3 for comparison purposes with other published studies, all data were log transformed for analysis unless otherwise indicated in figure legend or table legend. In the murine experiments, statistical significance (p ≤ 0.05) among treatment groups was determined via an unpaired t-test using GraphPad Prism (Prism 8, GraphPad Software, La Jolla, CA).

## Electronic supplementary material

Below is the link to the electronic supplementary material.

Supplementary Material 1

## Data Availability

The datasets used and/or analyzed during the current study are available from the corresponding author on reasonable request.
