# Supplementary material for: Novel nanoadjuvants balance immune activation with modest inflammation: implications for older adult vaccines
Source: Immun Ageing. 2023 Jun 21;20:28. doi: 10.1186/s12979-023-00349-5 (PMC10283283; doi:10.1186/s12979-023-00349-5)
Supplement: Supplementary file 1 — Supplementary Material 1 [file 12979_2023_349_MOESM1_ESM.docx]

Supplement Figure 1


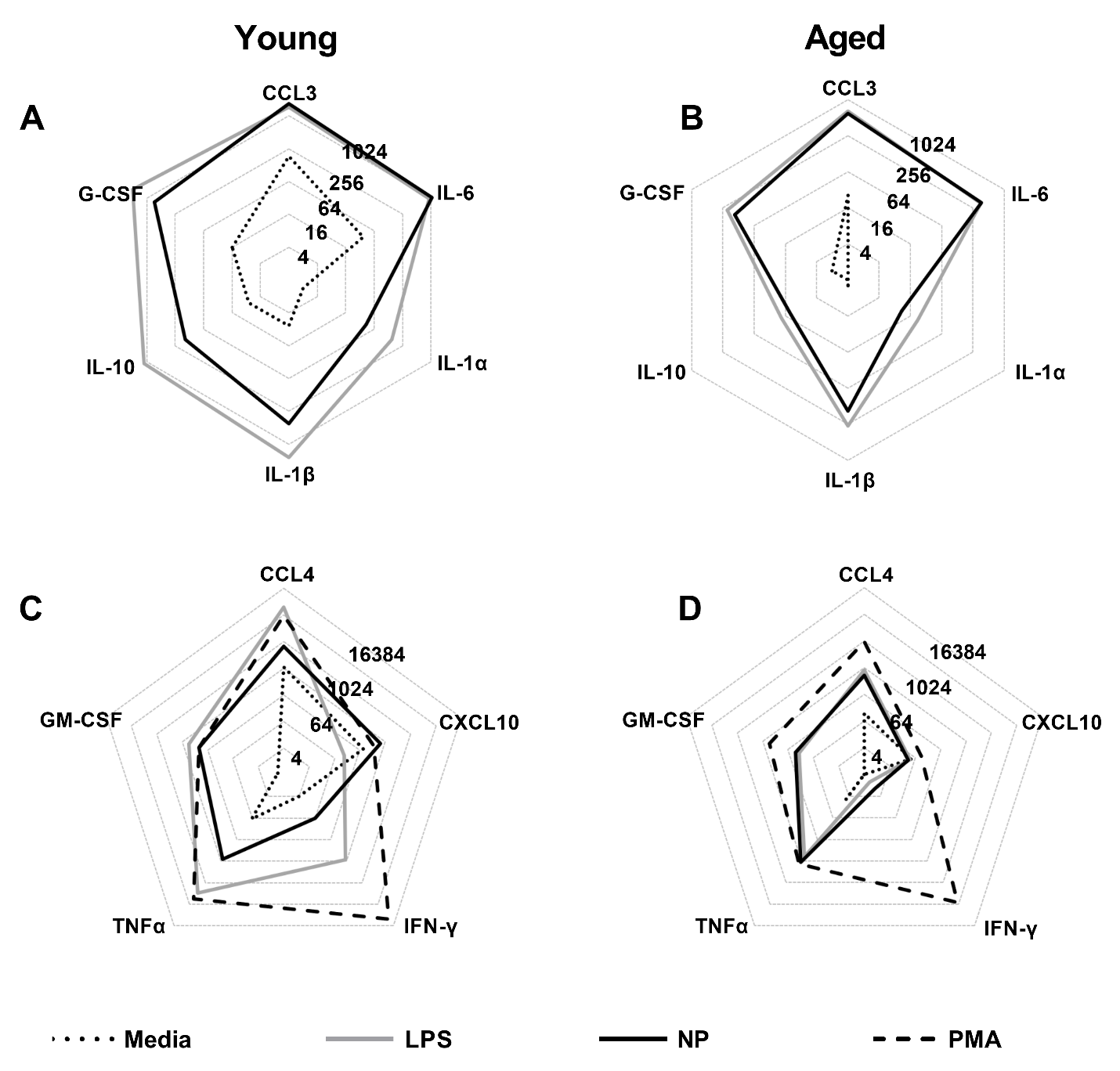


**Fig. S1** Cytokine or chemokine production by young or aged PBMCs is altered by NP, LPS, or PMA treatment. PBMCs from (A) young (n = 6) or (B) aged (n = 4) adults were incubated with either no adjuvants (media), polyanhydride nanoparticles (NP), LPS, or PMA/ionomycin (PMA) for 24 h and analyzed for production of cytokines, chemokines, or growth factors. A general pattern of response for CCL3, IL-6, IL-1α, IL-1β, IL-10, and G-CSF relative to media alone (log_2_ scale) is indicated A (young) or B (aged). The overall pattern indicates a more inflammatory profile for LPS compared to NP for this group of cytokine, chemokines, or growth factors. PBMCs from young (C) and aged (D) adults also responded to 24h treatment by NP, LPS or PMA resulting in changes of CCL4, CXCL10, IFN-γ, TNFα, and GM-CSF. The overall pattern indicated that NP tended to show reduced activation in comparison to PMA or LPS, yet the response to LPS varied by age or cytokine/chemokine.
